# Surgery of the amygdala and uncus: a case series of glioneuronal tumors

**DOI:** 10.1007/s00701-020-04249-1

**Published:** 2020-01-30

**Authors:** Andrew C. Vivas, Stephen Reintjes, Nir Shimony, Fernando L. Vale

**Affiliations:** 1 Department of Neurosurgery and Brain Repair, Morsani College of Medicine, University of South Florida, FL, Tampa, USA; 2 Johns Hopkins University School of Medicine, Institute for Brain Protection Sciences, Johns Hopkins All Children’s Hospital, Saint Petersburg, FL USA; 3 Geisinger Commonwealth School of Medicine, Neuroscience Institute, Scranton, PA USA; 4 Neurosurgery Department Lewis Katz School of medicine, Temple University, Philadelphia, PA USA; 5 Department of Neurosurgery, Medical College of Georgia, Augusta University, 1120 15th Street, BI 3088, Augusta, GA 30912 USA

**Keywords:** Amygdala, Uncus, Hippocampus sparing, Temporal lobe epilepsy, Low grade, Gliomas, Surgery, Neuropsychology

## Abstract

**Background:**

Patients with a lesion within the amygdala and uncus may develop temporal lobe epilepsy despite having functional mesial structures. Resection of functional hippocampus and surrounding structures may lead to unacceptable iatrogenic deficits. To our knowledge, there is limited descriptions of surgical techniques for selectively resecting the amygdala and uncus lesions while preserving the hippocampus in patients with language-dominant temporal lobe pathology.

**Methods:**

Thirteen patients with language-dominant temporal lobe epilepsy related to amygdala-centric lesions were identified. Patients with sclerosis of the mesial structures or evidence of pathology outside of the amygdala-uncus region were excluded. Neuropsychological evaluation confirmed normal function of the mesial structures ipsilateral to the lesion. All patients were worked up with video-EEG, high-resolution brain MRI, neuro-psychology evaluation, and either Wada or functional MRI testing.

**Results:**

All patients underwent selective resection of the lesion including amygdala and uncus with preservation of the hippocampus via a transcortical inferior temporal gyrus approach to the mesial temporal lobe. Pathology was compatible with glioneuronal tumors. Post-operative MRI demonstrated complete resection in all patients. Eight of the thirteen patients underwent post-operative neuropsychology evaluations and did not demonstrate any significant decline in tasks of delayed verbal recall or visual memory based on the Rey Auditory Verbal Learning Test (RAVLT). One patient showed a slight decrease in confrontation naming using the Boston Naming Test (BNT). Seizure freedom (Engel class I) was achieved in 12 of 13 patients.

**Conclusion:**

Selective transcortical amygdala and uncus resection with hippocampus preservation may be a reasonable way to achieve seizure control while sparing functional mesial structures.

**Electronic supplementary material:**

The online version of this article (10.1007/s00701-020-04249-1) contains supplementary material, which is available to authorized users.

## Introduction

Lesions at or near the amygdaloid nucleus commonly present with mesial temporal lobe epilepsy. Unlike patients with idiopathic epilepsy (i.e., non-lesional mesial temporal lobe epilepsy), lesional patients may have radiographically normal and functional hippocampus. Because the epileptic network in these patients is unrelated to the subjacent mesial temporal lobe, resection of the mesial structures may incur substantial neuropsychological decline if standard resection techniques are employed [6, 19–21]. However, most published data focuses on primary disease control and seizure freedom rather than functional outcomes.

Lesions of the amygdala are traditionally removed using an antero-lateral temporal approach. The traditional anterior temporal lobectomy (ATL) encompasses an en bloc resection of the anterior temporal lobe and mesial structures. Alternative “selective” corridors to reduce postsurgical neuropsychological deficits include (1) transsylvian [26], (2) subtemporal [11, 17], and (3) transcortical [4, 16] approaches. Each entails its own specific risks including vascular injury or vasospasm (transsylvian) [13], temporal lobe retraction and injury to the vein of Labbé (subtemporal), and the risk for resection of eloquent neocortex (transcortical) [15]. Regardless of the approach, most authors describe large craniotomies exposing the temporal lobe, Sylvian fissure, and frontal lobe [24].

Over the past decade, we have refined our technique for selective mesiobasal temporal resection of temporal lobe lesions. We perform a selective exposure using a small craniotomy with a trajectory through the inferior temporal gyrus (ITG) and remove the amygdala and surrounding archicortex, while preserving the hippocampus. The surgical technique and outcomes are presented. To our knowledge, this is the largest reported series of amydgala-centric lesional epilepsy surgery performed using only a limited access ITG approach.

## Methods

### Patient population

Lesional amygdalectomy was designed to spare the hippocampus in the dominant hemisphere of patients with lesions at the anterior temporal tip near or within the amygdaloid nucleus (amygdala-centric tumors). Patients had no clinical or radiographic evidence of mesial temporal sclerosis (MTS). All patients were selected based on the following criteria: MRI lesion at the level of the amygdala/uncus on the language-dominant left temporal lobe, normal MRI appearance of the hippocampus, intact memory functions in neuropsychological examination, and correlating electroencephalogram (EEG) findings of interictal and/or ictal epileptiform discharges in the anterior temporal electrodes. Language-dominant temporal lobe was defined using functional MRI (fMRI) or Wada testing. No high-grade gliomas were included in this dataset as well as vascular lesions. In addition, patients with lesions that extended into the brainstem, temporal stem, lateral basal ganglia, insular cortex, and superior or medial temporal gyrus were excluded.

A single surgical approach was used in all patients, i.e., transcortical access through the inferior temporal gyrus. Preoperative and postoperative MR images were obtained with a 1.5- or 3-T system with T1-weighted (with and without contrast) and T2-weighted sequences in all patients. Complications were documented. Gross confrontation visual field defects were recorded pre- and postoperatively, but formal visual field examinations were not routinely done.

### Operative technique and surgical anatomy

A similar operative technique for mesial temporal lobe lesions has been published previously [23]. There have been modifications tailored to the size and location of the lesions. The surgical intent is gross total resection of the tumor/lesion and removal of the amygdala, uncus, and entorhinal cortex while sparing the hippocampus.

**Fig. 1:**
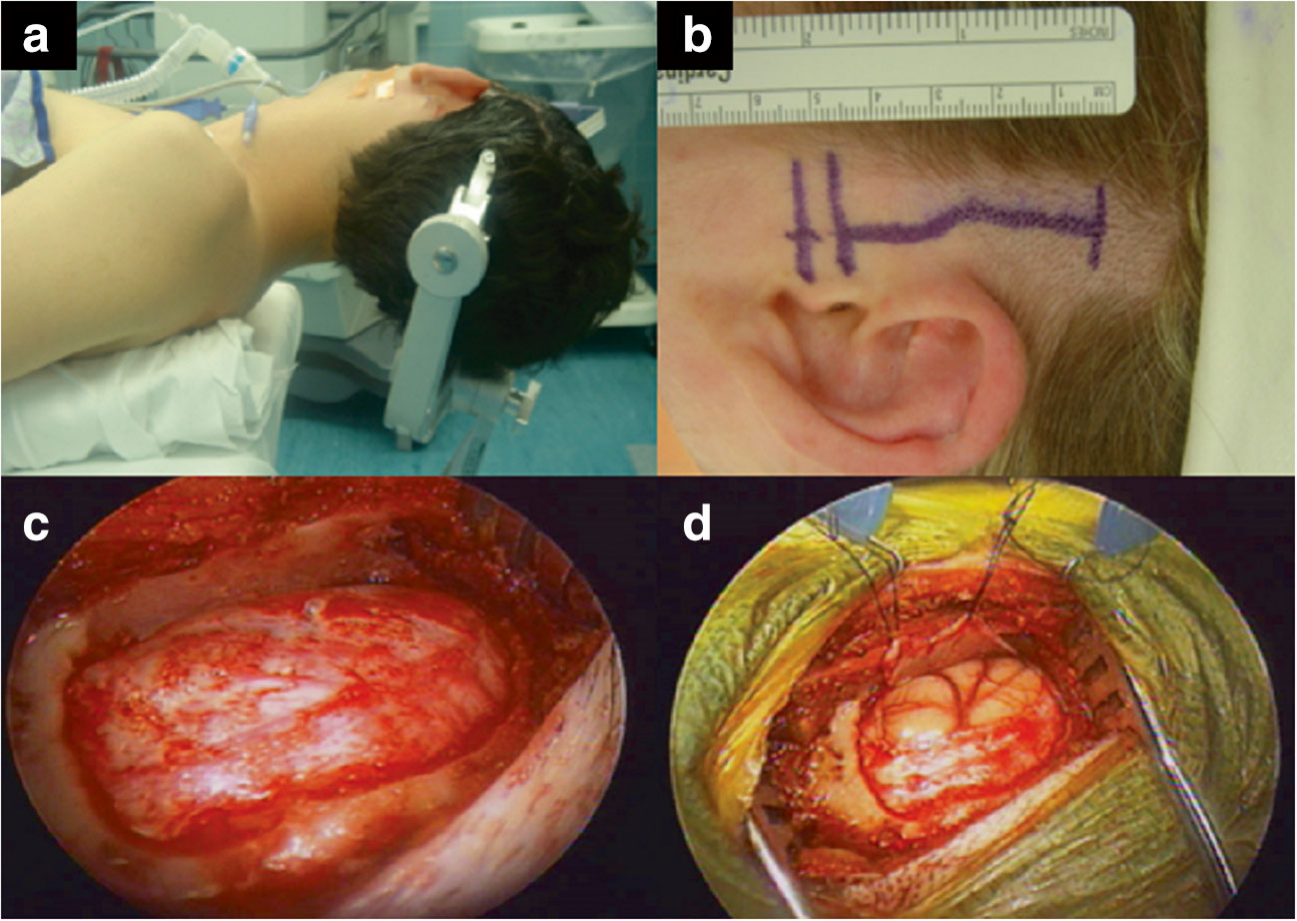
**a** Appropriate surgical positioning. The patient is positioned supine with a roll under the ipsilateral shoulder with the head turned almost to 90 degrees, with the neck extended to bring the zygoma up to be the most superior anatomical feature. **b** Typical incision length (approximately 6–8 cm) for a minimal access corridor to mesial structures. Care is taken not to expose the zygomatic arch by dissecting the temporalis muscle from its attachment to the bone (can lead to temporalis muscle atrophy). **c** Oval craniotomy based at the floor of the middle fossa to allow access to the temporal pole and temporal horn, without exposure of the Sylvian fissure or superior temporal gyrus. Frequently, a small amount of additional bone needs to be removed in order to ensure that the inferior aspect of the craniotomy is flush with the floor of the middle fossa. **d** C-shaped durotomy based on the floor of the middle fossa with the dura tacked up to improve visualization

Patients are placed in the supine position with a roll under the ipsilateral shoulder (Fig. 1a). A vertical linear incision is marked starting 5–10 mm in front of the inferior aspect of the tragus and extending superiorly approximately 6–8 cm (Fig. 1b). This produces an incision centered at the superior attachment of the auricle. An oval 2 × 3-cm craniotomy is then created (the wide portion is in the axial plane). In the coronal plane, the craniotomy center should be aligned with the root of the zygoma to maximize the exposure and allow appropriate access to the amygdala (Fig. 1c) [2]. Bone wax is applied if mastoid air cells are exposed. The dura is opened in a curvilinear fashion based on the floor of the middle fossa. The dural opening is limited to the inferior and lateral aspect of the temporal fossa (Fig. 1d). Brain relaxation techniques can be used to minimize brain retraction. Intraoperative image guidance helps to maintain precise spatial and anatomical orientation, delineating the margin between the tumor and the surrounding neural tissue. The intracranial dissection is then divided into 2 basic steps.

Step 1 involves resection of the ITG to provide a corridor to the mesial temporal lobe. Care is taken to stay within the ITG without disturbing the middle temporal gyrus (MTG). At this phase of the procedure, the operating table is placed in the Trendelenburg position to allow better visualization of the basal temporal lobe structures. The ITG corticectomy begins at the level of the zygomatic root, and the anterior extension is tailored accordingly (access corridor) to expose the temporal tip. For most cases, extending the resection of the ITG about 2.5 cm anteriorly from the zygomatic root will suffice. Landmarks encountered laterally to medially include the occipitotemporal sulcus, fusiform gyrus, and collateral sulcus. During the initial cortical resection, the trajectory of dissection should be parallel to the floor of the middle fossa, as dissection in a cephalad orientation will take the surgeon into the white matter of the temporal stem. Once the collateral sulcus is encountered, dissection in a 30–45-degree trajectory towards the vertex and slightly posterior will lead to the temporal horn. The temporal horn is opened at the most basal and anterior aspect of the ventricle to minimize disruption of the optic radiations. Drainage of cerebrospinal fluid from the ventricle allows further brain relaxation. Step 1 is complete when the tip of the temporal horn of the lateral ventricle is identified.

Step 2 entails isolation and resection of the amygdala (AMG), uncus, and anterior parahippocampal gyrus if necessary. Understanding the margins of the AMG is critical to achieving its resection, yet its precise spatial location is challenging to describe, as its maximal length and width are oriented at an oblique with respect to the axial, coronal, and sagittal planes. The AMG extends from the mesiobasal temporal lobe to the roof of the temporal horn, where it merges with the nucleus basalis and striatum. The dorsomedial border approximates the confluence of the hippocampal head and anterior uncus as the nucleus extends outward from the cisternal pial plane. Ventrolaterally, it is bounded by the inferior horn of the lateral ventricle [22].

**Fig. 2:**
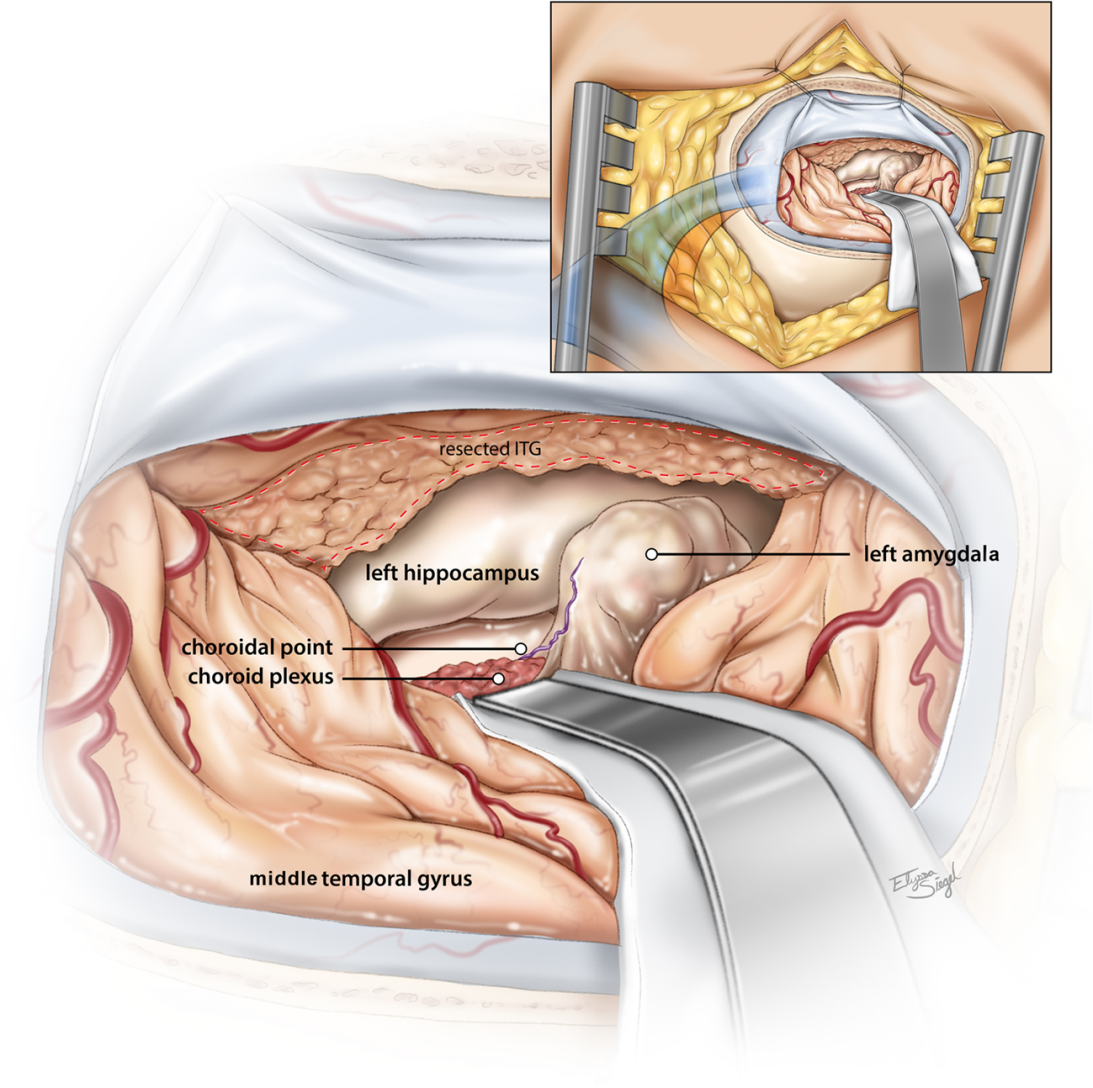
Artists rendition of the surgical exposure to the mesial structures via the inferior temporal gyrus approach (left side approach). The inferior temporal gyrus has been resected and the temporal horn opened. The choroid plexus can be seen posteriorly as a marker of the posterior border of the amygdala. Within the temporal horn, constituting the mesial aspect of the ventricle is the hippocampus. The head of the hippocampus can be disconnected from its anterolateral border from the adjacent amygdala

Microsurgical resection of the AMG begins with opening the temporal horn to identify the choroid plexus, choroidal fissure, and anterior hippocampus, defining the anatomical limits of the AMG (Fig. 2). The rostral plane between the AMG and the roof of the temporal horn is often delineated by a vein that lies along the margin between the two structures. The superior extent of resection is limited to the level of the roof of the temporal horn to avoid entering the nucleus basalis and striatum rostrally. The choroid plexus is identified within the temporal horn and is useful as a surrogate for the anterior choroidal artery (and the choroidal point). An imaginary line connecting the choroidal point to the proximal MCA bifurcation demarcates the dorsal margin of the AMG (thru an intact pial plane). The AMG can be disconnected and mobilized anteriorly. The dorsomesial limit is achieved by disconnecting the nucleus from the confluence of the anterior hippocampus and uncus. This resection is carried mesially until the pial plane of the basal cisterns is encountered. Great care is taken to dissect the mesial temporal structures in a subpial fashion (provides protection from entering the crural and ambient cisterns). Identification of the optic tract if visible (thru an intact pial plane) is an additional reference point for the superomesial extent of the AMG. Once the mesial dissection has been achieved and the crural cistern is exposed, the third nerve, proximal internal cerebral artery (ICA), posterior cerebral artery (PCA), and brainstem will be in view. (Video 1). Once the AMG has been dissected free along the ventral border (connecting the initial ventrolateral dissection to the ventromedial dissection), a 360-degree resection has been achieved and the nucleus/lesion can be removed en bloc if necessary. Bipolar coagulation of the mesial arachnoid surface should be avoided. Bleeding from the mesial pia-arachnoid should be controlled with hemostatic agents such as oxidized cellulose polymer. Removal of the uncus can help to better delineate the borders of the AMG, but it is not a necessary step and can be accomplished before or after resecting the lesion. An anterior parahippocampectomy is performed at this stage (tailored to the lesion).

## Results

Between 2007 and 2017, 123 patients with temporal lobe lesions were identified and received treatment at our institution after referral to the Comprehensive Epilepsy Center. Thirty-two patients had isolated lesions within the anterior temporal lobe. In eighteen patients, MRI demonstrated dominant temporal lobe lesions (all left-sided pathology) at or near the amygdala and uncus with no lateral cortex involvement and no evidence of MTS/hippocampus involvement. Patients presented with new-onset seizures or suspected epileptic events. All surgeries were performed by the senior author. Patient information was obtained retrospectively from the operative and epilepsy surgery databases after approval from the Institutional Review Board. All patients provided informed consent.

Thirteen patients were diagnosed with glioneuronal tumors of the anterior mesial-basal temporal lobe (uncus and/or amygdala) (see Table 1). No gross visual field loss was identified preoperatively on confrontation testing. Normal pre-operative memory function was documented based on neuro-psychology testing in all patients according to protocol [18]. Language dominance was determined using Wada testing in 8 patients and more recently, five patients using fMRI.

**Table 1:** Patient’s demographics (all left-sided surgery)

	Age, sex	Pathology	Seizure outcome
1	22, M	DNET	SF
2	17, M	Ganglioglioma	NSF
3	62,F	PXA	SF
4	42, M	DNET	SF
5	13, M	Ganglioglioma	SF
6	47, F	DNET	SF
7	23, M	DNET	SF
8	25, F	DNET	SF
9	48, F	DNET	SF
10	39, M	DNET	SF
11	38, M	DNET	SF
12	54, F	PXA	SF
13	16, M	Ganglioglioma	SF

*SF*, seizure-free; *NSF*, not seizure-free; *DNET*, dysembryoplastic neuroepithelial tumor; *PXA*, pleomorphic xanthoastrocytoma

**Fig. 3:**
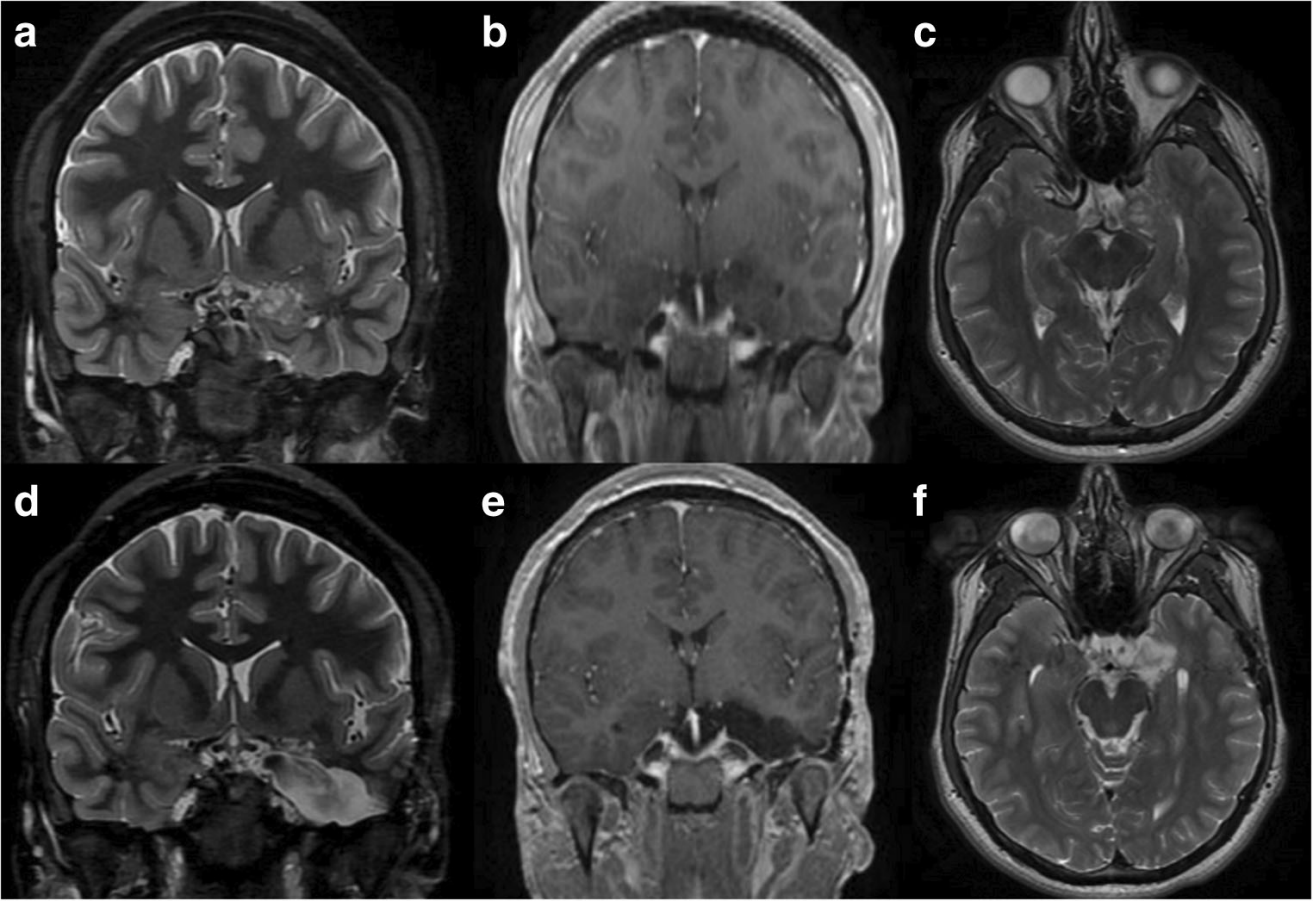
**a**–**c** Preoperative coronal T2, T1 with gadolinium, and axial T2 series reveal a non-enhancing lesion within the mesial structures of the temporal lobe. **d**–**e** Postoperative coronal T2, T1 with gadolinium, and axial T2 series reveal a limited access trajectory with resection of the amygdalar region with sparing of the posterior hippocampus

Post-operative MRI demonstrated complete lesion (including amygdala and uncus) resection in all patients (Fig. 3). Eight patients had post-operative neuropsychology evaluation which failed to demonstrate a significant decline in tasks of delayed verbal recall and visual memory based on the Rey Auditory Verbal Learning Test (RAVLT). One patient showed a slight decrease in confrontation naming using the Boston Naming Test (BNT). Unfortunately, five patients did not undergo postoperative psychology evaluation due to financial limitations, insurance denial, or personal reasons. No patients were lost to follow-up.

Complete seizure control was documented in 12 of the 13 patients (92%) (Table 1). The average length of follow-up was 36 months (range 20–60 months). No gross visual field loss was identified post-operatively on confrontation testing. There were no major permanent neurological deficits, medical complications, or deaths.

## Discussion

The amygdala or amygdaloid nucleus was initially described by Karl Friedrich Burdach in a three-volume treatise entitled Vom Baue und Leben des Gehirns (1819–1826) [3]. It is a structurally diverse complex of about 13 nuclei that are functionally related to the olfactory and limbic systems. The AMG also contains the nucleus of origin of the stria terminalis*.* Yet, it has no well-defined surgical borders. Surgical identification of intra-operative landmarks decreases the risk of injury to the surrounding structures and afford surgeons the ability to perform en bloc resection of the lesion(s) for detailed histopathologic analysis.

Feindel and Rasmussen reported the first attempt to resect the entire amygdala with minimal hippocampus resection [8]. This pivotal study demonstrated the importance of the amygdala and anterior temporal lobe structures in seizure generation and sparked the interest in more selective surgical interventions. Their results prove to be efficacious in epilepsy surgery. This approach, while technically challenging, is somewhat easier to understand for surgeons who have epilepsy surgery experience. The goal in this series is to tailor resection to the preoperative MRI lesion. Resection of these structures include the amygdala, the uncus, and the most anterior part of the parahippocampal gyrus. The role of the amygdala in the epileptogenecity of the temporal lobe is well documented [7]. The rationale of this approach is the disruption of the mesial temporal epileptic network or lesion with an attempt to preserve functional memory outcome.

Safe and complete resection of the amygdala and surrounding structures is the main objective. In this technique, access corridor using a small corticectomy (ITG) allows exposure of the ventricle without brain retraction. Because the AMG blends with the white matter tracts, globus pallidus and anterior commissure, it is recommended to use the roof of the ventricle as a landmark for the superior extent of the resection. A limited exposure of the choroid plexus is helpful for anatomic orientation and marks the posterior border of exposure. Other authors have suggested using the choroidal point as a surgical landmark in the resection of the amygdala [25]. We do not advocate exposing the anterior choroidal point however, as this risks inadvertent injury to the anterior choroidal artery. This complex intraoperative anatomy was elegantly described by Tubbs et al [22]. An imaginary line connecting the roof of the ventricle with the proximal MCA bifurcation will allow complete or near complete safe resection of the amygdala.

Apical temporal lobe resections (extended cortico-amygdalectomy) have been used to spare the dominant hippocampus in patients with MRI lesions at the apex of the temporal lobe without clinical or radiographic evidence of mesial sclerosis [5, 14]. Elsharkawy et.al demonstrated good long-term seizure control and worsening memory performance in 3.2% of patients. However, the authors did not provide any information with regard to naming assessment for these patients [5]. It is known that more extended neocortical resections involving the middle and superior temporal gyri carry a risk of worsening naming difficulties [9]. Our current surgical approach seems to preserve memory function while minimizing the risk of naming difficulties seen with larger neocortical resections.

Patients with a normal hippocampus have a higher risk of post-operative deficit even with selective resections [10, 12, 18]. The epileptogenic focus in well-defined lesions (such as those with certain low grade gliomas and cavernous malformations) is understood to be within the MRI visible portion of the lesion and its immediate surroundings [1]. As such, adequate seizure control can be achieved by selective lesionectomy. Resection of the normal hippocampus offers risk of neuropsychological decline without necessarily added seizure control. Memory preservation has been previously documented with adequate seizure control in patients with significant medically resistant epilepsy [12].

In this review, we demonstrate the safety and efficacy of selective lesional amygdalectomy, yet no claims are made of its originality. It is a compromise between a standard ATL and more selective techniques. Comparison of this technique to others is challenging due to the constant evolution of surgical technology, patient selection, and surgical protocols. Subtle neuropsychological deterioration or visual field deficit is only detectable after extensive post-operative testing, which was not performed in all patients. Lastly, detailed knowledge of microsurgical anatomy is crucial for successful minimal access surgery, and there may be a steep learning curve for surgeons unfamiliar with this technique.

## Conclusions

Surgical approaches to the amygdala and uncus have been redefined as we begin to understand its intricate association with language, speech, memory, and vision. Lesional amygdalectomy associated with long-term epilepsy was initially designed to spare the hippocampus in the dominant hemisphere of patients with lesions near the amygdaloid nucleus. Despite the challenges of mesial basal temporal lobe surgery, this approach provides adequate and safe exposure for selective amygdala and uncus resection in either temporal lobe for a variety of surgical indications.

## Electronic supplementary material

Video 1 Operative video demonstrates preserved hippocampus medially being separated from the amygdala anteriorly. The lateral ventricle is open and the choroid plexus can be seen posteriorly as a marker of the anterior border of the amygdala. At the completion of the resection the hippocampus can be seen, preserved. The amygdala, with the associated uncus has been resected with preservation of the mesial pial plane separating the ambient cistern, third cranial nerve, PCA, and midbrain from the middle fossa structures. (MP4 79,880 kb)
